# Timely Initiation of Breastfeeding and Its Associated Factors at the Public Health Facilities of Dire Dawa City, Eastern Ethiopia, 2021

**DOI:** 10.1155/2022/2974396

**Published:** 2022-09-06

**Authors:** Natnael Dechasa Gemeda, Fentahun Alemnew Chekole, Wondu Feyisa Balcha, Nigusu Ayalew Gessesse

**Affiliations:** ^1^Dire Dawa University, School of Medicine and Health Sciences, Department of Midwifery, Dire Dawa, Ethiopia; ^2^Bahir Dar University, College of Medicine and Health Sciences, Department of Midwifery, Bahir Dar, Ethiopia

## Abstract

**Introduction:**

Timely initiation of breastfeeding is defined as putting the newborn baby to the breast within one hour of birth. Despite the World Health Organization and national recommendations on timely initiation of breastfeeding, delayed initiation of breastfeeding is still a common problem.

**Objective:**

The aim of this study was to assess the timely initiation of breastfeeding and its associated factors at the public health facilities of Dire Dawa city, Eastern Ethiopia, 2021.

**Methods:**

A health facility-based cross-sectional study was employed from February 1, 2021, to March 2, 2021, at the public health facilities of Dire Dawa city among 302 mother-child pairs. The data were collected by systematic random sampling technique, entered into Epi data 4.2, and analyzed using Statistical Package of Social Science 25.0 version. Bivariate and multivariable logistic regression analyses were employed to estimate the crude and adjusted odds ratio with a confidence interval of 95%, and a *P* value of < 0.05 was considered statistically significant. Frequency tables, figures, and descriptive summaries were used to describe the study variables.

**Results:**

In this study, timely initiation of breastfeeding was 70.9% (95% CI: 65.6-75.8%). In a multivariable analysis, maternal age group of 25-40 years (AOR = 2.21, 95% CI = 1.09 − 4.48), multiparty (AOR = 2.58, 95% CI = 1.24 − 5.40), counselling on timely initiation of breastfeeding during antenatal care visits (AOR = 2.38, 95% CI = 1.16 − 4.88), institutional delivery (AOR = 3.29, 95% CI = 1.27 − 8.52), vaginal delivery (AOR = 3.06, 95% CI = 1.20 − 7.81), counselling on breastfeeding immediately after delivery (AOR = 2.89, 95% CI = 1.29 − 6.45), not practicing pre lacteal feeding (AOR = 6.76, 95% CI = 2.35 − 19.44), and having good practice of colostrum feeding (AOR = 4.03, 95% CI = 1.95 − 8.36) were associated with timely initiation of breastfeeding. *Conclusion and Recommendation.* Mothers who had practiced timely initiation of breastfeeding were low compared to the national recommendation (92%). Age of the mother, multiparity, counseling on timely initiation of breastfeeding, institutional delivery, vaginal delivery, counseling after delivery, not practicing prelacteal feeding, and having a good practice of colostrum feeding were predictors of timely initiation of breastfeeding. It indicates a need to encourage mothers to have antenatal care visits and institutional delivery.

## 1. Background

The first hours and days after birth are one of the riskiest periods of a child's life, but getting an early start to breastfeeding offers a powerful line of defences [1]. Babies are born ready to breastfeed; however, breastfeeding was an endangered practice in both rich and poor countries [2]. Breastfeeding is an unequalled way of providing ideal food for the healthy growth and development of infants; it is also an integral part of the reproductive process with important implications for the health of mothers [2].

When breastfeeding is delayed after birth, the consequences can be life-threatening and the longer newborns are left waiting, the greater the risk. For newborns, every minute counts, waiting 2-23 hours increases their risk of death by 1.3 times, and waiting 1 day or more increases their risk of death by more than 2 times [1]. Globally, about 2.6 million neonates die each year, most of which occurred within the first 7 days after birth, with about 1 million dying on the first day and close to 1 million dying within the next 6 days [3]. Two-thirds of neonatal mortality occur in South-East Asia and Sub-Saharan Africa [4, 5].

Based on the World Health Organization (WHO) report, globally over one million newborn infants could be saved each year by initiating breastfeeding within the first hour of life [6]. Timely initiation of breastfeeding (TIBF) is defined as putting the newborn baby to the breast within one hour of birth [1]. Studies reported that TIBF can reduce more than 20% of neonatal deaths [7]. The Baby-Friendly Hospital Initiative develops ten steps to successful breastfeeding and has been accepted as minimum global criteria for designation of a hospital as Baby-Friendly. TIBF is one of the ten steps of successful breastfeeding [8].

Timely initiation of breastfeeding is recognized as the first and vital step toward reducing mortality in infants and children under five years of age [9]. The WHO and the United Nations Children's Fund recommend that breastfeeding should be initiated within the first hour after birth, continued exclusively for the first 6 months of life, and continued, with safe and adequate complementary foods, up to 2 years or beyond [3, 8].

An estimated about 78 million newborns had to wait more than one hour to be put to the breast and only 42% of newborns were put to the breast within the first hour of life, ranging 35% in the Middle East and North Africa to 65% in Eastern and Southern Africa [1]. Furthermore, no country had more than 80% of babies breastfeeding within an hour of birth [10]. In Ethiopia, according to EDHS 2016 report, the prevalence of TIBF was 73% [11].

Even though the WHO and the United Nations Children's Fund recommended initiating breastfeeding within the first hour of birth, still a large number of mothers practiced delayed initiation of breastfeeding [3, 12]. In developing countries like Ethiopia, delayed initiation of breastfeeding, discarding colostrum, and the introduction of dirty and unsound artificial feeding of infants with very dilute milk products are common [2]. Consequently, these man-made problems affect directly and indirectly the health of newborn infants and cause malnutrition and increase the risk of infection and death among neonates [7, 13, 14].

The Sustainable Development Goals target (SDG), to reduce neonatal and under-five deaths to less than 12, and 25 per 1000 live births respectively through eliminating preventable child deaths by the year 2030[15]. Knowing the practice of TIBF is important for the implementation of the SDG. Our study was conducted on mothers who gave birth of mature and healthy newborn, while preterm birth was excluded.

## 2. Methods

### 2.1. Study Design and Period

A health facility-based cross-sectional study design was employed from February 1, 2021, to March 2, 2021, at the public health facilities of Dire Dawa city.

### 2.2. Study Area

The study was conducted in Dire Dawa city health facilities. It is located about 515 km away from the capital city of Ethiopia, Addis Ababa. The city has ten public health facilities (two hospitals and eight health centers). The city has a total population of 506,936, of these 248,298 are males and 258,638 are females. There were about 162,220 rural and 344,716 urban residents with an average household size of 4.5 and 2.5, respectively [16].

### 2.3. Source Population

The source population was mothers who were visiting the child immunization clinic at the public health facilities of Dire Dawa city.

### 2.4. Study Population

The study population was all mothers who attended the child immunization clinic in selected public health facilities of Dire Dawa city during the study period.

### 2.5. Inclusion and Exclusion Criteria

All mothers who had babies less than or equal to twelve months and attended the child immunization clinic at the selected public health facilities of Dire Dawa city were included, while mothers who gave preterm birth excluded.

### 2.6. Study Variables

#### 2.6.1. Dependent Variable

Timely initiation of breastfeeding: the mother was asked whether she have ever breastfed, and then TIBF was assigned with a code of “1” if she started breastfeeding within one hour of delivery and “0” if she started breastfeeding after one hour of delivery)

#### 2.6.2. Independent Variables

Sociodemographic factors: age, residency, marital status, religion, educational level, occupation, partner educational status, and with whom she was living

Obstetrics factors: parity, ANC visit, counseling on TIBF, colostrum feeding and breastfeeding during ANC visits, place of delivery, birth attendant, mode of delivery, and counseling on breastfeeding immediately after delivery.

Knowledge and practice of colostrum and breastfeeding-related factors

#### 2.6.3. Operational Definitions

Timely initiation of breastfeeding: is defined as putting the neonate on the mother's breast to suckle breast milk (or colostrum) within one hour of birth as reported by the mother [17]

Colostrum: it is the yellowish breast milk produced within the first few days after delivery [18]

Practice: the behaviour, habit, or custom of mothers of infants on colostrum feeding of their current infants. Mothers were considered to have a good practice of colostrum feeding if she correctly answered ≥60% of the total practice assessing questions

Knowledge: refers to the knowledge of mothers about colostrum and breastfeeding and includes the timing of initiation of breastfeeding and an awareness and understanding of the mothers about the advantage of colostrum feeding. It was evaluated by the mother's answer to the knowledge-related questions. The mother was considered to have good knowledge if she correctly answered ≥60% of the total knowledge assessing questions [17]

#### 2.6.4. Sample Size Determination

The sample size was calculated using a single population proportion formula by considering the following assumptions: the proportion of TIBF among mothers having children less than two years in Debre Tabor was 76.8% [19], Z*α*/2 = critical value for normal distribution at 95% confidence level, which is equal to 1.96 (Z value of alpha = 0.05) or 5% level of significance (*α* = 0.05) and a 5% margin of error (*ω* = 0.05).

(1) $$\begin{array}{c} \text{Sample size }\left(n\right)=\frac{{\left(Z\alpha /2\right)}^{2}p\;\left(1-p\right)}{d2\;},n=\frac{\left(1.96\right)20.768\left(1-0.768\right)}{\left(0.05\right)2}=274 \end{array}$$

The sample size was adjusted by adding a 10% nonresponse rate, and the final sample size was 302 mother-child pairs.

#### 2.6.5. Sampling Procedure and Technique

The data were collected in the selected public health facility of Dire Dawa city. The total sample size was proportionally allocated for each health facility of the city based on their monthly expanded program of immunization unit flow. The average number of mothers who visited the expanded program of immunization unit per month based on the expanded program of immunization registration book at all selected health facilities was 1405. The numbers of mothers who visited the expanded program of immunization unit monthly were 570, 310, 251, and 274 in Dill Chora referral hospital, Genda kore health center, Gende Gerada health center, and Goro health center, respectively. The total sample size was proportionally allocated for each health facility, based on their population size, and by using the following formula;

(2) $$\begin{array}{c} \text{Sample in the health facility}=\text{total sample }\left(n\right)\text{ x }\frac{\text{population in the health facility }\left(Ni\right)}{\text{total population of the facilities }\left(N\right)} \end{array}$$

The total sample size after proportional allocation was 122, 67, 54, and 59 mothers, respectively, in Dill Chora referral hospital, Genda kore health center, Gende Gerada health center, and Goro health center. Eligible mothers in each facility were selected by using systematic random sampling techniques. The sampling interval or the *K*^th^ units (1405/302 = 5) were obtained by dividing the numbers of mothers who visited the expanded program of immunization unit monthly by the sample size. The starting unit was selected by using the lottery method among the first *K*^th^ units in each health facility.

#### 2.6.6. Data Collection Tools and Procedures

A structured interviewer-administered questionnaire was used to collect the data which were adapted from relevant works of literature and modified to the local context. Questionnaires were first prepared in the English language, and then, it was translated into Amharic by an individual who has good ability of these languages and then retranslated back into English to check the consistency. The questionnaire consisted of sociodemographic characteristics, reproductive, knowledge, and practice of colostrum and breastfeeding-related questions. Knowledge and practice of breastfeeding questions were assessed by +1 for a correct answer and 0 for an incorrect answer. The score for each mother was summed and categorized. A pretested structured interviewer-administered questionnaire was used for data collection purposes. The data were collected by four BSc midwives and supervised by one public health officer.

#### 2.6.7. Data Quality Control

Data were collected by trained data collectors, and pretesting of the instrument was done before the actual data collection. The questionnaire was pretested before the actual data collection period on 5% [13] mothers who attended a child immunization clinic in Sabian General Hospital, which is not selected in this study. Data collectors and the supervisors were trained for two days by the investigator. After necessary modifications and correction were done to standardize and ensure its reliability and validity, additional adjustments were made based on the results of the pretest. The completeness of the data was checked by data collectors during data collection, and daily supervision was done for data completeness by supervisors.

#### 2.6.8. Data Processing, Analysis, and Interpretation

The data were entered into Epi data 4.2, edited and cleaned for inconsistencies, missing values, and outliers, and then exported to SPSS version 25.0 for analysis. During analysis, all explanatory variables which have a significant association in bivariate analysis with a *P* value < 0.20 were entered into a multivariable logistic regression model to get AOR, and those variables with 95% of CI and a *P* value of < 0.05 were considered as statistically significant with TIBF. The multicollinearity test was done using variance inflation factor, and there was collinearity between the place of delivery and birth attendant. But, after removing birth attendants, there was no collinearity exists between the independent variables. The model goodness of the test was checked by using Hosmer-Lemeshow goodness of the fit, and its *P* value was 0.176. Frequency tables, figures, and descriptive summaries were used to describe the study variables.

## 3. Results

### 3.1. Sociodemographic Characteristics of the Mothers

A total of 302 mothers participated in the study with a response rate of 100%. The mean age of the mothers was 24.86 years with (±SD = 4.62) ranging from 15 to -40 years. Of these, 138 (45.7%) were found in the age group of 20-25 years. About, 80% (n = 241) of the mothers lives in urban and 287 (95.0%) were married. Of the mothers, 157 (52.0%) were Muslim religious followers and 117 (38.7%) had primary educational levels. More than half (57.0%) were housewives and 260 (86.1%) lives with their partner (Table 1).

### 3.2. Obstetric Characteristics of the Mothers

In this study, 222 (73.5%) of the mothers were multigravida and 245 (81.1%) had a history of ANC visit in their most recent pregnancy. Among mothers who had a history of ANC visits, 198 (80.8%) were counseled on TIBF, and 196 (80.0%) were counseled on EBF. Of the mothers, 258 (85.4%) gave childbirth at a health institution and 261 (86.4%) of the childbirth were attended by health care professionals. Nearly, 87% (*n* = 262) of the mothers had a history of vaginal delivery and 240 (79.5%) were counseled on breastfeeding immediately after delivery (Table 2).

### 3.3. Knowledge of the Mothers on Colostrum and Breastfeeding

In this study, according to the predetermined criteria, 189 (62.6%) mothers had good knowledge of colostrum and breastfeeding. About half, 152 (50.3%), of the mothers obtained their information on colostrum and breastfeeding from health care professionals. Of the mothers, 269 (89.1%) responded that colostrum is the mother's breast milk during the first three days of delivery and 296 (98.0%) knew that colostrum is yellow in color. About 71% (*n* = 215) of the mothers responded that breastfeeding should start within an hour after delivery, and 163 (54.0%) knew that colostrum is nutritious and hygienic. Of the mothers, 207 (68.5%) responded that the baby should feed colostrum and breast milk on demand day and night without provision of any prelacteal feeding (PLF), and 157 (52.0%) knew that colostrum is important for the growth and development of the baby. About 210 (69.5%) responded that the baby should feed breast milk even if the mother is sick, and 238 (78.8%) of the mothers responded that the baby should feed breast when he/she is sick (Table 3).

### 3.4. The Practice of Colostrum Feeding

Two-thirds of the mothers, 206 (68.2%), had good practice of colostrum feeding. Of the mothers, 268 (88.7%) gave colostrum to their index child immediately after birth. It may cause abdominal discomfort 9 (26.5%) and my breast has no milk 9 (26.5%) were their main reason for not feeding colostrum immediately after delivering. PLF was practiced by 47 (15.6%) of the mothers. The cultural practice was responded by 17 (36.2%) of the mothers as a reason of practicing PLF, and 20 (42.6%) were given infant formula milk (Table 4).

### 3.5. Timely Initiation of Breastfeeding

**Figure 1:**
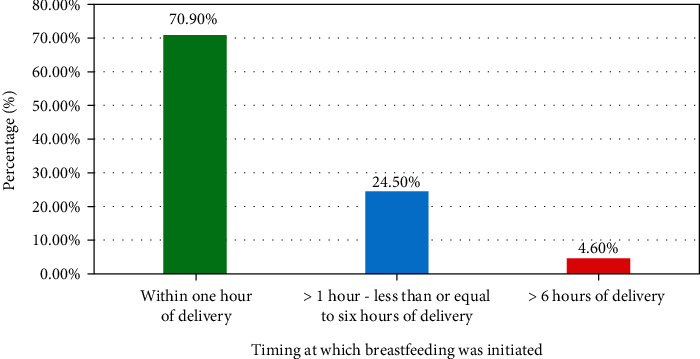
Timing at which breastfeeding was initiated among mothers who attended the child immunization clinic in the public health facilities of Dire Dawa city, Eastern Ethiopia, 2021 (*n* = 302).

In our study, 214 (70.9%) with [95% CI: 65.6-75.8%] of the mothers initiate breastfeeding timely (within one hour of delivery) (Figure 1).

### 3.6. Association between Timely Initiation of Breastfeeding and Prelacteal Feeding

**Figure 2:**
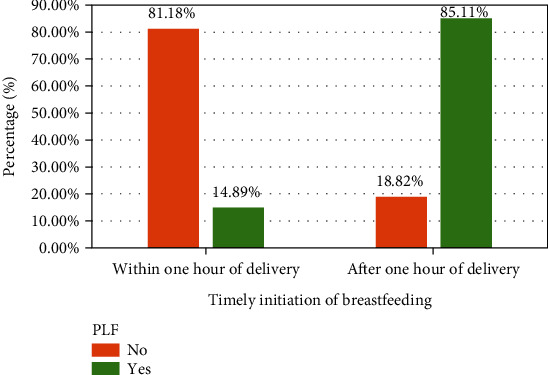
Association between TIBF and PLF among mothers who attended the child immunization clinic in the public health facilities of Dire Dawa city, Eastern Ethiopia, 2021 (*n* = 302).

There was a significant association between TIBF and PLF at a *P* value < 0.001. Among mothers who practiced TIBF, 207 (81.18%) were not given PLF to their index child, while mothers who started breastfeeding after one hour of delivery 40 (85.11%) were given PLF to their index child (Figure 2).

### 3.7. Factors Associated with Timely Initiation of Breastfeeding

In bivariate analysis, maternal age, residency, educational level of the mothers, parity, history of ANC visit, counseling on; TIBF, and EBF during ANC visits, place of delivery, mode of delivery, counseling on breastfeeding immediately after delivery, not practicing PLF, good practice of colostrum feeding, and knowledge on colostrum and breastfeeding were significantly associated with TIBF at a *P* value of < 0.20. In a multivariable logistic regression analysis, maternal age, parity, counseling on TIBF during ANC visits, place of delivery, mode of delivery, counseling on breastfeeding immediately after delivery, not practicing PLF, and good practice of colostrum feeding remained significantly associated with TIBF at a *P* value of < 0.05.

Mothers who are found in the age group of 25-40 years were 2.21 times more likely to practice TIBF than mothers who are found in the age group of 15-24 years (AOR = 2.21, 95% CI = 1.09 − 4.48), and multiparous mothers were 2.58 times more likely to initiate breastfeeding early relative to primipara mothers (AOR = 2.58, 95% CI = 1.24 − 5.40). Mothers who are counseled on TIBF during their ANC visits were 2.38 times more likely to practice TIBF relative to those who are not counseled on TIBF (AOR = 2.38, 95% CI = 1.16 − 4.88), and giving childbirth at health institution was increasing the chance of TIBF by 3.39 relative to mothers who gave birth at home (AOR = 3.29, 95% CI = 1.27 − 8.52). Mothers who had a history of vaginal delivery were 3.06 times more likely to initiate breastfeeding early relative to mothers who gave birth by caesarean section (AOR = 3.06, 95% CI = 1.20 − 7.81), and counseling on breastfeeding immediately after delivery increased the chance of TIBF by 2.89 compared to mothers who are not counseled on breastfeeding immediately after delivery (AOR = 2.89, 95% CI = 1.29 − 6.45). Mothers who do not practice PLF were 6.76 times more likely to practice TIBF than those who practiced PLF (AOR = 6.76, 95% CI = 2.35 − 19.44), and having a good practice of colostrum feeding was increasing the chance of TIBF by 4.03 relative to mothers who had a poor practice of colostrum feeding (AOR = 4.03, 95% CI = 1.95 − 8.36) (Table 5).

## 4. Discussion

In our study, TIBF was practiced by 70.9% of the mothers with [95% CI of 65.6-75.8%]. This finding was in line with the 2016 EDHS report (73.0%) [11]. It was also in line with studies conducted in the Dembecha district (73.1%) [17] and Arsi zone (67.3%) [20], as well as with a study conducted in Uganda (68.6%) [21]. However, it was lower than studies conducted in different parts of Ethiopia like Bedessa town (81.1%) [22], North Wollo (78.2%) [23], Bahir Dar city (87.0%) [24], Gunchire town (80.5%) [25], Western Ethiopia (88.5%) [26], Dale Woreda (83.7%) [27], Motta town (78.8%) [28], Debre Tabor (76.8%) [19], Mekelle town (77.9%) [29], South Gondar Zone hospitals (88.2%) [30], and Wolaita Sodo City (80.2%) [31]. The probable explanation for the discrepancy between our study and the aforementioned studies might be due to the difference in the studies' sitting, as the majority of the above-mentioned study conducted in town sitting and the majority of mothers who lived in town sitting had formal education, while our study includes both urban and rural residency. There is supporting evidence from studies that shows that mothers who are lived in urban and had formal education were more likely to practice TIBF than their counterparts [32–35].

The result in our study was also lower than studies conducted in Australia on women who were born in Turkey, Australia, and Vietnam and gave childbirth in Australia showing that the rate of TIBF was 8%, 84%, and 75%, respectively [36], Saudi Arabia (77.8%) [37], and south of Iran (96.0%) [38]. This discrepancy might be attributed to the difference in sociodemographic characteristics of the study participants and the implementation of health system.

The finding in this study was higher than the studies done in Ethiopia like Mizan-Aman town (64.5%) [39], Axum town (41.6%) [40], Goba Woreda (52.4%) [34], Benishangul Gumuz (53.8%) [41], Arba Minch Zuria (57.2%) [42], rural eastern zone of Tigray region (61.9%) [43], Debre Berhan town (62.6%) [44], South Gonder zone (48.7%) [45], Amibara district (39.6%) [33], Gurage zone (43.7%) [32], Jimma Arjo Woreda (63.0%) [46], and rural pastoralist communities of Afar region (63.6%) [47]. The possible reason might be the studies' sitting; for instance, the studies conducted in Arba Minch Zuria, Jimma Arjo Woreda, and rural pastoralist communities of Afar have included only a rural residence, while this study includes both urban (80.0%) and rural residency (20.0%). The other probable reason might be a time gap of the studies as utilization of maternal and child health services increased through time and this may help the mothers to get information about the advantage of TIBF in the form of health education or counseling. The practice of TIBF was also higher than studies conducted in Tanzania (51.0%) [48], Nigeria (45%) [49], Turkey (35.2%) [50], Brazil (47.1%) [51], and India (36.4%) [52]. The probable reason for this discrepancy might be due to the cultural difference in breastfeeding practices of the countries.

In this study, sociodemographic and obstetrics characteristics and practice and knowledge level of mothers on colostrum and breastfeeding were significantly associated with TIBF. Mothers who are found in the age group of 25-40 were 2.21 times more likely to practice TIBF. The possible explanation might be mothers who are found in the age group of greater than or equal to 25 years may have more previous experience of breastfeeding as seen in this study more than three-fourth of women who are found in the age group of greater than or equal to 25 were multiparous. There is supporting evidence from studies conducted in different countries showing that being older-aged mothers were more likely to practice TIBF [37, 53].

Multipara mothers were 2.58 times more likely to practice TIBF. This finding was in line with other studies [35, 37, 45, 53]. The possible reason might be having previous experience of childbirth make multiparous mothers start breastfeeding earlier than primipara mothers. The other possible explanation might be multiparous mothers may have good skills and knowledge of newborn care and proper infant feeding practice. Mothers who are counseled on TIBF during their ANC visits were 2.38 times more likely to practice TIBF. This finding was supported by other studies conducted in Ethiopia [31, 54]. The possible reason might be getting counseling about the advantage of early starting of breastfeeding may make them start breastfeeding timely, and this intern may increase their chance of practicing optimal breastfeeding.

Giving childbirth at the health institution increases the chance of TIBF by 3.29 times. This was consistent with other studies [25, 28, 45, 47, 53, 55–57]. The possible reason might be that giving childbirth at the health institution with the assistance of a health care provider may increase their chance of getting counseling about the advantage of TIBF compared to those who delivered at home with the assistance of a traditional birth attendant. The other possible explanation for this could be a difference in the health education provided by health professionals as part of labor and delivery care, and immediately after childbirth when compared with traditional birth attendants. There is supporting evidence from different studies conducted in Ethiopia showing that mothers who were attended by a traditional birth attendant/relatives during their last childbirth were less likely to practice TIBF [17, 20, 44].

Mothers who gave childbirth vaginally were 3.06 times more likely to initiate breastfeeding early. This was supported by studies conducted in Wolaita Sodo city [31], South Gonder zone [45], Gunchire town [25], Bahir Dar city [24], rural Eastern zone of Tigray region [43], and Motta town [28]. This was also consistent with studies conducted in Sub-Saharan Africa and India [52, 53]. The 2016 EDHS secondary data analysis also shows that mothers who gave birth by cesarean section were 86% less likely to start breastfeeding early compared to mothers who had a vaginal delivery [55]. Mothers who gave birth by cesarean section were less likely to practice TIBF [33, 44, 58].

Counseling on breastfeeding immediately after delivery increased the chance of TIBF by 2.29 times. The possible reason might be being counseled after delivery may increase their chance of getting information about the advantage of TIBF over delayed initiation of breastfeeding. There is supporting evidence from studies conducted in Ethiopia [31, 54]. Mothers who do not practice PLF were 6.76 times more likely to practice TIBF. This result was in line with another study [28]. The possible reason might be mothers who do not practice PLF may have good knowledge about breastfeeding. This was supported by studies conducted in Ethiopia showing that mothers who had good knowledge of breastfeeding were more likely to initiate breastfeeding timely [24, 25].

Having a good practice of colostrum feeding increased the chance of TIBF by 4.03 times. The possible explanation might be mothers who had a poor practice of colostrum may take time to discard the first best and nutritious baby breast milk and this may delay the time of initiating breastfeeding. There is supporting evidence from a study conducted in Debre Berhan showing that not feeding colostrum was associated with delayed initiation of breastfeeding [44]. Delayed initiation of breastfeeding could be due to an attempt to discard colostrum, because milking or pumping out the colostrum may take more times even a day until it is removed from the breast and white milk starts to come out.

## 5. Limitation of the Study

Since this study included mothers whose index child age was up 12 months, recall bias might have occurred. To avoid this recall bias, we tried to remind them to remember the condition at the time of the delivery by asking questions like how was your feeling at the time of delivering and immediately after delivery, who was with you on that day, and when do you touch the body of your baby with your hands.

## 6. Conclusion and Recommendations

In our study, TIBF was comparable when compared to the 2016 EDHS report (73.0%), but it was lower when compared to the nationally recommended level of TIBF (92%). Among the predictors, age of the mother, counselled on TIBF during ANC visit, multiparty, institutional delivery, vaginal delivery, counseling on breastfeeding immediately after delivery, not practicing PLF, and having a good practice on colostrum and breastfeeding were significantly associated with TIBF. Even if in our study more than two-third of mothers practiced TIBF, still its gap is wide with WHO and national recommendation of breastfeeding. Therefore, massive awareness creation on the advantage of TIBF and avoidance of malpractices such as practicing PLF and delayed initiation of breastfeeding is needed. As well as promoting ANC visit and institutional delivery for all pregnant women is recommended for the increasing of TIBF. Therefore, to enable mothers to establish and sustain TIBF, it is crucial to increase a mother's level of knowledge of infant and young child feeding, as a cornerstone for implementing sustainable strategies to improve appropriate feeding practices.

## Figures and Tables

**Table 1:** Sociodemographic characteristics of the mothers who attended the child immunization clinic in the public health facilities of Dire Dawa city, Eastern Ethiopia, 2021 (*n* = 302).

Variables	No. (%)
Maternal age in years	
15-19	20 (6.6)
20-25	138 (45.7)
36-30	110 (36.4)
≥31	34 (11.3)
Residence	
Rural	62 (20.3)
Urban	244 (79.7)
Religion	
Muslim	157 (52.0)
Orthodox	132 (43.7)
Others^∗^	13 (4.3)
Marital status	
Married	287 (95.0)
Others^∗∗^	15 (5.0)
Maternal educational level	
Had no formal education	50 (16.6)
Primary education	117 (38.7)
Secondary education	78 (25.8)
Diploma and above	57 (18.9)
Maternal occupational status	
Housewife	172 (57.0)
Merchants	80 (26.5)
Employed	50 (16.5)
Partner educational level (*n* = 287)	
No formal education	37 (12.9)
Primary education	82 (28.6)
Secondary education	89 (30.0)
Diploma and above	79 (27.5)
Mother living with	
Partner	260 (86.1)
With other else	42 (13.9)

^∗^Protestant and Catholic. ^∗∗^Single, divorced, and widowed.

**Table 2:** Obstetric characteristics of the mothers who attended the child immunization clinic in the public health facilities of Dire Dawa city, Eastern Ethiopia, 2021 (*n* = 302).

Variables	No. (%)
Parity	
Primipara	80 (26.5)
Multipara	222 (73.5)
History of ANC visit	
Yes	245 (81.1)
No	57 (18.9)
Counseled on TIBF during ANC visits (*n* = 245)	
Yes	198 (80.8)
No	47 (19.2)
Counseled on EBF during ANC visits (*n* = 245)	
Yes	196 (80.0)
No	49 (20.0)
Place of delivery	
Home	44 (14.6)
Health institution	258 (85.4)
Mood of delivery	
Vaginal delivery	262 (86.8)
Cesarean section	40 (13.2)
Birth attendants	
Health care professionals	261 (86.4)
Traditional birth attendants/family	41 (13.6)
Counseled on breastfeeding immediately after delivery	
Yes	240 (79.5)
No	62 (20.5)

**Table 3:** Knowledge on colostrum and breastfeeding among mothers who attended the child immunization clinic in the public health facilities of Dire Dawa city, Eastern Ethiopia, 2021 (*n* = 302).

Variables	No. (%)
Source of information	
Health professional	152 (50.3)
Mass media	73 (24.2)
Family/friends	77 (25.5)
Colostrum is the mother breast milk during the first three days of delivery	
Yes	269 (89.1)
No	33 (10.9)
Color of colostrum	
Yellow	296 (98.0)
White	6 (2.0)
Breastfeeding should be started within an hour after delivery	
Yes	215 (71.2)
No	87 (28.8)
Colostrum is nutritious and hygienic	
Yes	163 (54.0)
No	139 (46.0)
Colostrum is the best first milk given to the baby	
Yes	250 (82.8)
No	52 (17.2)
Timely initiation of breastfeeding strengthens baby-mother bonding	
Yes	282 (93.4)
No	20 (6.6)
Early initiation of breastfeeding prevents breast pain/engorgement after birth	
Yes	172 (57.0)
No	130 (43.0)
Early initiation of breastfeeding prevents vaginal bleeding after birth	
Yes	142 (47.0)
No	160 (53.0)
The baby should feed colostrum and breast milk on demand day and night	
Yes	207 (68.5)
No	95 (31.5)
Colostrum important for the growth and development of the baby	
Yes	157 (52.0)
No	145 (48.0)
Colostrum gives natural immunity to the baby	
Yes	111 (36.8)
No	191 (63.2)
Should child feed breast when the mother is sick	
Yes	210 (69.5)
No	92 (30.5)
Should child feed breast when he/she is sick	
Yes	238 (78.8)
No	64 (21.2)
Colostrum protects the newborn from diseases	
Yes	103 (34.1)
No	199 (65.9)
Knowledge on colostrum and breastfeeding	
Good knowledge	189 (62.6)
Poor knowledge	113 (37.4)

**Table 4:** Colostrum feeding practice among mothers who attended the child immunization clinic in the public health facilities of Dire Dawa city, Eastern Ethiopia, 2021 (*n* = 302).

Variables	No. (%)
Did you feed colostrum to the baby immediately after birth	
Yes	268 (88.7)
No	34 (11.3)
If no reason for not feeding colostrum (*n* = 34)	
It causes abdominal discomfort and diarrhea	9 (26.5)
My breast has no milk	9 (26.5)
Colostrum is not clean	8 (23.5)
Baby unable to suck	5 (14.7)
I was sick	4 (11.8)
Prelacteal feeding	
Yes	47 (15.6)
No	255 (84.4)
Reason for pre lacteal feeding (*n* = 47)	
It is a cultural practice	17 (36.2)
Not having enough milk	16 (34.0)
Breast pain	10 (21.3)
I was sick	4 (8.5)
Types of pre lacteal feeding (*n* = 47)	
Formula milk	20 (42.6)
Cow milk	11 (23.4)
Plain water	7 (14.9)
Sugar solution	5 (10.6)
Honey	4 (8.5)
Did you give the baby breast milk within the first three days after delivery	
Yes	296 (98.0)
No	6 (2.0)
Did you put the baby to the breast immediately after delivery	
Yes	220 (72.8)
No	82 (27.2)
The practice of colostrum feeding	
Good practice of colostrum feeding	206 (68.2)
Poor practice of colostrum feeding	96 (31.8)

**Table 5:** Logistic regression analysis for TIBF among mothers who attended the child immunization clinic in the public health facilities of Dire Dawa city, Eastern Ethiopia, 2021 (*n* = 302).

Variables	TIBF		COR (95% CI)	AOR (95% CI)	*P* value
	Yes	No			
Maternal age in years					
15-24	102	56	1	1	
25-40	112	32	1.92 (1.15-3.20)	2.21 (1.09-4.48)	0.028^∗^
Residency					
Rural	32	29	1	1	
Urban	182	59	2.80 (1.56-5.00)	1.16 (0.50-2.73)	0.725
Maternal educational level					
No formal	27	23	1	1	
education	81	36	1.92 (0.97-	1.22 (0.43-	0.711
Primary	106	29	3.79)	3.44)	0.559
education secondary and above			3.11 (1.56-6.22)	1.35 (0.49-3.74)	
Parity					
Primipara	40	40	1	1	
Multipara	174	48	3.62 (2.11-6.23)	2.58 (1.24-5.40)	0.012^∗^
History of ANC visits					
No	15	42	1	1	
Yes	199	46	12.11 (1.19-23.70)	0.85 (0.23-3.09)	0.805
Counseled on TIBF					
No	47	77	1	1	
Yes	167	31	6.53 (3.79-11.26)	2.38 (1.16-4.88)	0.018^∗^
Counseled on EBF					
No	48	58	1	1	
Yes	166	30	6.69 (3.87-11.53)	1.58 (0.68-3.66)	0.283
Place of delivery					
Home	16	28	1	1	
Health institution	198	60	5.77 (2.93-11.38)	3.29 (1.27-8.52)	0.014^∗^
Mode of delivery					
Cesarean section	17	23	1	1	
SVD	197	65	4.10 (2.06-8.15)	3.06 (1.20-7.81)	0.019^∗^
Counseled on breastfeeding immediately after delivery					
No	27	35	1	1	
Yes	187	53	4.57 (2.54-8.23)	2.89 (1.29-6.45)	0.010^∗^
Pre lacteal feeding					
Yes	7	40	1	1	
No	207	48	24.64 (10.41-58.36)	6.76 (2.35-19.44)	0.001^∗^
Practice of colostrum feeding					
Poor practice	36	60	1	1	
Good practice	178	28	10.59 (5.97-18.81)	4.03 (1.95-8.36)	0.001^∗^
Knowledge of breastfeeding					
Poor knowledge	63	50	1	1	
Good knowledge	151	38	3.15 (1.89-5.27)	1.31 (0.63-2.75)	0.470

^∗^Significant at a *P* value of < 0.05.

## Data Availability

All related data have been presented within the manuscript. The data set supporting the conclusion of this article is available from the corresponding author upon reasonable request.

## Abbreviations

ANC: Antenatal care
AOR: Adjusted odd ratio
CI: Confidence interval
COR: Crude odds ratio
EBF: Exclusive breastfeeding
EDHS: Ethiopian demographic health survey
PLF: Prelacteal feeding
SDG: Sustainable Development Goals
TIBF: Timely initiation of breast feeding
WHO: World Health Organization.
